# LncRNA SLCO4A1-AS1 Accelerates Growth and Metastasis of Gastric Cancer *via* Regulation of the miR-149/XIAP Axis

**DOI:** 10.3389/fonc.2021.683256

**Published:** 2021-09-28

**Authors:** Yantian Fang, Bo Sun, Jianpeng Gao, Yakai Huang, Chenchen Wang

**Affiliations:** ^1^ Department of Gastric Surgery, Fudan University Shanghai Cancer Center, Shanghai, China; ^2^ Department of Oncology, Shanghai Medical College, Fudan University, Shanghai, China; ^3^ Department of Medical Oncology, Fudan University Shanghai Cancer Center, Shanghai, China

**Keywords:** LncRNA SLCO4A1-AS1, gastric cancer, miR-149, X-linked inhibitor of apoptosis, metastasis

## Abstract

**Objective:**

Recently, long noncoding RNA SLCO4A1 antisense RNA 1 (SLCO4A1-AS1) has been shown to act as an oncogene in several cancer types; however, its role in gastric cancer (GC) and its underlying molecular mechanisms are yet to be elucidated.

**Methods:**

Using the ENCORI database, we identified SLCO4A1-AS1, miR-149-5p (miR-149), and the X-linked inhibitor of apoptosis (XIAP) whose expressions were obviously changed in GC samples, and analyzed the correlation between their expressions in GC samples. Moreover, we explored the expression of SLCO4A1-AS1, miR-149, and XIAP in clinical samples and GC cell lines using RT-qPCR and western blotting assay; the correlation between them was analyzed using RNA immunoprecipitation and dual-luciferase reporter. CCK-8, colony formation, and Transwell assays were conducted to determine the effects of SLCO4A1-AS1, miR-149, and XIAP expression on cell proliferation, migration, and invasion, respectively. A nude mouse xenograft model was used to explore their function in xenograft growth.

**Results:**

SLCO4A1-AS1 was significantly upregulated in the GC samples and cell lines, and a high level of SLCO4A1-AS1 was associated with an advanced tumor stage and shortened patient survival. Mechanistically, SLCO4A1-AS1 post-transcriptionally regulated XIAP by functioning as competing endogenous RNA in GC to sponge miR-149. Further functional assays revealed that the overexpression of miR-149 and knockdown of XIAP considerably inhibited GC cell viability and its migratory and invasive characteristics *in vitro*. SLCO4A1-AS1 knockdown also determined the function of GC cells but was diminished by the miR-149 inhibitor *in vitro*. Finally, we demonstrated that the deletion of SLCO4A1-AS1 suppressed tumor growth and metastasis *in vivo*.

**Conclusions:**

Altogether, these findings suggest that SLCO4A1-AS1 functions as a crucial oncogenic lncRNA in GC and it can facilitate GC tumor growth and metastasis by interacting with miR-149 and enhancing XIAP expression. Therefore, SLCO4A1-AS1 is a potential novel therapeutic target in GC treatment.

## Introduction

Gastric cancer (GC) is the fourth most frequently diagnosed malignancy that as a high mortality due to its high recurrence rate and distant metastasis (1, 2). The lack of symptoms in the early stages of GC means that most patients are diagnosed at the advanced disease stage or after distant metastases has occurred (3). Although significant progress has been made in the diagnosis and treatment strategies for GC, the prognosis for patients in the advanced stage remains poor (4). Therefore, efforts to develop new biological targets for the diagnosis and treatment of GC are ongoing.

Long noncoding RNAs (lncRNAs) are noncoding RNAs (>200 nt) that are dysregulated in several types of cancer and involved in the regulation of various cellular processes, such as cell proliferation, apoptosis, and invasion (5, 6). In recent years, new evidence has revealed that some lncRNAs act as oncogenic or tumor suppressor genes in GC. For example, LINC00682 suppresses GC progression through the modulation of the microRNA-9-LMX1A axis (7); UCA1 facilitates GC cell proliferation and migration by suppressing p21 and SPRY1 levels (8); and KRT19P3 represses GC metastasis through the regulation of COPS7A-mediated NF-κB signaling (9). It has been reported that lncRNA SLCO4A1-AS1 functions as an oncogenic gene in colorectal and bladder cancer (10, 11). Nevertheless, the biological functions and underlying mechanisms of SLCO4A1-AS1 in GC are yet to be determined.

Several studies have reported that certain lncRNAs may function as competitive endogenous RNAs (ceRNA) by binding to miRNA response elements upregulating mRNAs and participating in tumorigenesis and development (12). SLCO4A1-AS1 acts as a sponge for miR-508-3p and increases PARD3 expression and encourages colorectal cancer progression (13), whereas SLCO4A1-AS1 accelerates bladder cancer cell invasion by functioning as miR-335-5p ceRNA to promote OCT4 expression (11). We speculated that SLCO4A1-AS1 is also be involved in GC development through a similar mechanism.

In this study, we showed that SLCO4A1-AS1 was markedly upregulated in GC samples and cell lines. SLCO4A1-AS1 post-transcriptionally regulated XIAP by acting as a ceRNA in GC to sponge miR-149-5p (miR-149). SLCO4A1-AS1 plays an oncogenic role in GC progression by interacting with miR-149 and enhancing XIAP expression.

## Materials and Methods

### GC Samples and Cell Lines

In all, 88 GC and paired noncancerous samples were obtained from patients with GC undergoing radical surgery at the Fudan University Shanghai Cancer Center. Approval was granted by the Committee for Ethical Review of Research involving Fudan University Shanghai Cancer Center, and all patients gave their informed consent. Four human GC cell lines, MKN45, AGS, NCI-N87, and SGC-7901, and a noncancerous gastric cell line, GES-1, were acquired from the American Type Culture Collection (Manassas, VA, USA) and maintained in RPMI-1640 medium supplied with 10% fetal bovine serum (FBS).

### Oligonucleotides and Transfection

miR-149 mimic and inhibitor and their negative controls were purchased from GenePharma (Shanghai, China). SLCO4A1-AS1 siRNA (5’-GCCTGAGCTTGTTCACAAA-3’) and XIAP siRNA (5’-GTGGTAGTCCTGTTTCAGC-3’) were chemically synthesized by Sangon Biotech (Shanghai, China). SLCO4A1-AS1 knockdown and control lentiviruses were obtained from Hanbio (Shanghai, China). The GC cells were infected with the lentiviruses to obtain stable SLCO4A1-AS1 knockdown and overexpressing cells. The oligonucleotides were then transfected into GC cells with Lipofectamine 2000 (Invitrogen, Carlsbad, CA, USA).

### RNA Isolation and Real-Time Quantitative PCR

The TRIzol Reagent was used to isolate total RNAs from the cell or tissue samples. Cytoplasmic and nuclear RNAs were extracted using Thermo Fisher BioReagents (Thermo Fisher Scientific, USA) according to the manufacturer’s instructions. cDNA was synthesized using the PrimeScript RT Reagent Kit (TaKaRa, Dalian, China). The Applied Biosystems 7500 Sequence Detection System was used to conduct real-time quantitative PCR (RT-qPCR) using the SYBR Green PCR Master Mix (Applied Biosystems, Foster City, CA, USA). The lncRNA and mRNA expression were normalized to *GAPDH* levels. The PCR primers for SLCO4A1-AS1 were 5’-GAGTGTCGCTGACTTGAA-3’ and 5’-CCGTCTGTTCCTGATTCTT-3’, those for *XIAP* were 5’- AATAGTGCCACGCAGTCTACA-3’ and 5’-CAGATGGCCTGTCTAAGGCAA-3’, and those for *GAPDH* were 5’-AGCCACATCGCTCAGACAC-3’ and 5’- GCCCAATACGACCAAATCC-3’. The miRNA levels were determined using Stem-Loop Primer SYBR Green Quantitative Real-time-PCR (RiboBio, Guangzhou, China), and the expression was normalized to *U6* levels. Standard curves were generated and the 2^−△△CT^ method was used.

### RNA Pull-Down Assay

SLCO4A1-AS1-overexpressing MKN45 cells were fixed by 1% formaldehyde, lysed, and sonicated. The supernatant was incubated with a SLCO4A1-AS1-specific probesstreptavidin dynabeads (M-280; Invitrogen) mixture overnight. After washing, the RNA was extracted for further detection.

### Western Blot Assay

SDS-PAGE was performed to separate the proteins which were then transferred to PVDF and subsequently incubated with primary antibodies against XIAP and GAPDH (Cell Signaling Technology, Beverly, MA, USA). The membranes were then washed and incubated with a specific secondary antibody, and ECL Blotting Detection Reagents were used to visualize the specific bands.

### Dual-Luciferase Reporter Assay

Plasmids were constructed using fragments of XIAP 3′UTR and SLCO4A1-AS1, including the binding site of miR-149 or the fragment carrying the mutant binding site for miR-149, and inserted into the pmirGLO vector. The miR-149 mimic (negative control) and luciferase reporter plasmids were cotransfected into the GC cells. Plasmids with mut-binding sites were used as controls. Luciferase activity was measured using a dual-luciferase reporter assay system (Promega, Madison, WI, USA).

### RNA Immunoprecipitation

Magna RIP™ RNA-Binding Protein Immunoprecipitation Kit (Millipore, Burlington, MA, USA) was used to perform RNA immunoprecipitation (RIP) experiments. MKN45 cells transfected with miR-149 mimic or inhibitor were lysed and the cell lysates were centrifuged. The supernatant was added to a RIP immunoprecipitation buffer that included either AGO2-conjugated or IgG-conjugated magnetic beads. The retrieved RNA was detected using RT-qPCR.

### Cell Proliferation Analysis

Cell viability was determined using Cell Counting Kit-8 (CCK-8, Dojindo, Japan). Briefly, GC cells were plated onto 96-well plates with 3000 cells/well. Cell proliferation capacity was evaluated at 1, 2, 3, 4, and 5 days after transfection by detecting the absorbance at 490 nm using a plate reader. Colony formation assay was conducted using infected MKN45 and AGS cells that were seeded onto six-well plates with 400 cells/well for 12 days, fixed, stained, and counted under a microscope.

### Cell Migration and Invasion Assays

A 24-well plate was used to conduct cell migration and invasion assays. Cells were plated into the upper chamber wells with a non-coated membrane (Millipore) for Transwell migration assays and Matrigel-coated membrane for Transwell invasion assays. RPMI-1640 without FBS was used to resuspend the cells and was placed in the upper chamber wells, whereas RPMI-1640 with 10% FBS was moved into the lower chamber wells. After incubation for 24 h, cells that migrated through the membrane were stained with 4% paraformaldehyde for 15 min followed by 0.1% crystal violet for 60 min. Cell imaging was done using an inverted microscope and ImageJ software was used to quantify the cells.

### *In Vivo* Assays

Animal manipulation experiments were performed after approval by the Institutional Animal Care and Use Committee of the Fudan University Shanghai Cancer Center. Immunodeficient BABL/c female nude mice (4-week-old) were obtained and kept under disease-free conditions. For the tumor formation assay, 3 × 10^6^ MKN45 cells infected with lentivirus were injected into the right dorsal flank of the mice (n = 5). Tumor volume was determined every 5 days, and the tumors were acquired and weighed 30 days after injection. For the tumor metastasis assay, 1 × 10^6^ MKN45 cells were injected into the mice *via* the tail vein. After 50 days, the mice were sacrificed and their lungs were obtained for HE staining. The images of the lungs were captured and the number of metastatic nodules was counted.

### Immunohistochemical Staining

The tumor tissues obtained from the different groups were fixed, embedded, and cut into 4 μm slices. After deparaffinization, the slices were rehydrated and subjected to antigen retrieval and treated with antibodies against Ki67, XIAP, and CD31 (Cell Signaling Technology).

### Statistical Analysis

All results of the experimental analyses are presented as the mean  ± standard deviation, and the data were analyzed using Student’s *t*-test or analysis of variance. The correlations among miR-149, SLCO4A1-AS1, and XIAP were analyzed using Spearman rank correlation analysis. All analyses were performed using GraphPad Prism 5.0 (GraphPad Prism Software, GraphPad, San Diego, CA, USA), and the statistical significance was set at *P* < 0.05.

## Results

### SLCO4A1-AS1 Expression Was Upregulated in GC Tissues and Cell Lines

**Figure 1:**
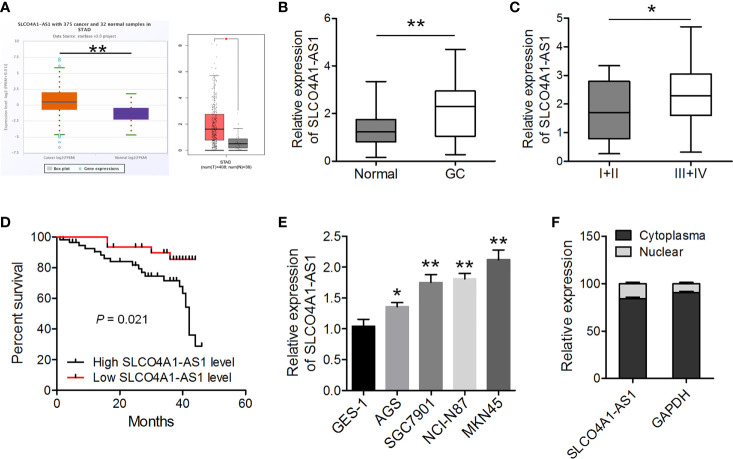
SLCO4A1-AS1 expression increased in gastric cancer (GC) tissues and cell lines. **(A)** SLCO4A1-AS1 expression in GC and normal samples in the ENCORI and GEPIA databases. **(B)** SLCO4A1-AS1 expression was measured using RT-qPCR in GC and paired normal tissues from 88 patients with GC. **(C)** SLCO4A1-AS1 expression in association with clinical stages of gastric tumor samples. **(D)** The overall survival curves of the 88 patients with GC with high SLCO4A1-AS1 and low SLCO4A1-AS1 expressions. **(E)** RT-qPCR analysis for SLCO4A1-AS1 levels in GC and GES-1 cells. **(F)** RT-qPCR data indicating the abundance of SLCO4A1-AS1 in either the cytoplasm or nucleus of AGS cells. GAPDH was used as internal control for cytoplasmic RNA. ^*^ *P* < 0.05, ^**^ *P* < 0.01.

First, we determined the levels of SLCO4A1-AS1 in GC samples and normal samples using the ENCORI (http://starbase.sysu.edu.cn/panCancer.php) and GEPIA (http://gepia.cancer-pku.cn/) databases. The results indicated that the levels were higher in the GC samples than in the normal samples (**Figure 1A**). The expression of SLCO4A1-AS1 in GC was subsequently detected in 88 GC tissues and paired normal tissues using RT-qPCR. In 64% (57/88) of the patients with GC, SLCO4A1-AS1 expression in tissues had increased compared with that in normal tissues; this was consistent with the ENCORI results (**Figure 1B**). The clinical and pathological characteristics of the patients with GC were then classified into two groups on the basis of median SLCO4A1-AS1 expression. Notably, a high SLCO4A1-AS1 expression was positively correlated with advanced TNM stages (**Figure 1C** and **Table 1**). Notably, increased SLCO4A1-AS1 expression was strongly associated with shorter patient survival (**Figure 1D**). Consistently, RT-qPCR results showed higher SLCO4A1-AS1 expression in the GC cell lines than in the normal GES-1 cell line (**Figure 1E**). To demonstrate the cellular localization of SLCO4A1-AS1, we conducted RT-qPCR analysis of nuclear and cytoplasmic SLCO4A1-AS1 RNA; the results demonstrated that SLCO4A1-AS1 preferentially localized in the cytoplasm (**Figure 1F**). Taken together, these findings showed that SLCO4A1-AS1 expression was enhanced in GC and may act as an oncogenic gene in GC progression.

**Table 1:** Correlative analysis of SLCO4A1-AS1 levels with different clinical features of gastric cancers.

Variable	SLCO4A1-AS1 expression		*χ^2^*	*P-value
	High expression	Low expression		
Age			0.006	0.938
>55	40	22		
≤55	17	9		
Gender			0.531	0.466
Male	40	24		
Female	17	7		
Tumor size (cm^3^)			2.098	0.148
>35	10	2		
≤35	47	29		
Stage			5.159	0.023
I+II	14	15		
III+IV	43	16		
Distant metastases			2.279	0.131
M0	53	31		
M1	4	0		
Degree of differentiation			0.358	0.550
Well and moderately	22	14		
Poorly	35	17		

^*^P < 0.05 by χ^2^ test.

### SLCO4A1-AS1 Acts as a Sponge for miR-149 in GC

**Figure 2:**
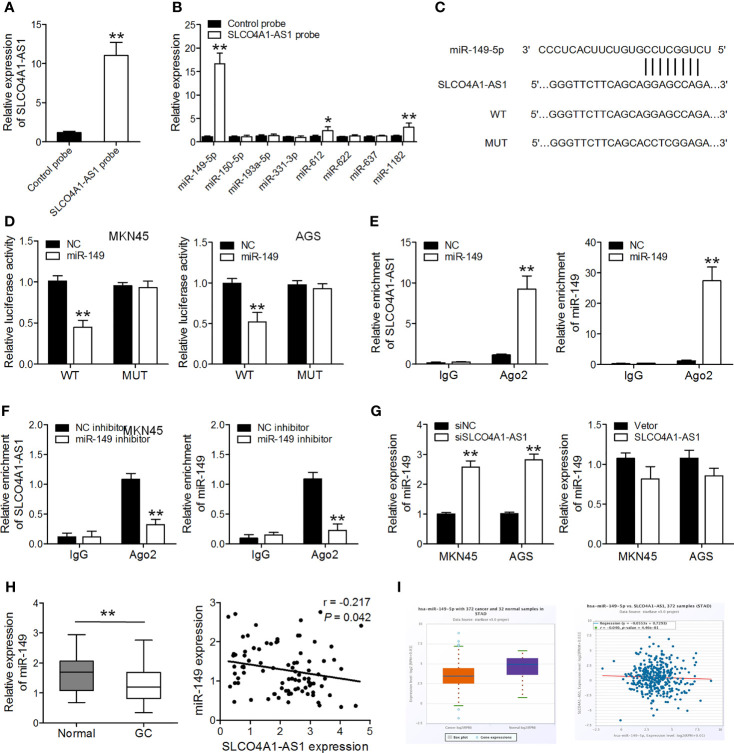
SLCO4A1-AS1 serves as a sponge for miR-149 in gastric cancer (GC). **(A)** SLCO4A1-AS1 in MKN45 cell lysis was pulled down and enriched with SLCO4A1-AS1 specific probe and then assessed by RT-qPCR. **(B)** miRNAs in MKN45 cell lysis was pulled down and enriched with SLCO4A1-AS1 specific probe and then assessed by RT-qPCR. **(C)** Sequence alignment of SLCO4A1-AS1 with wild-type (WT) *versus* mutant (MUT) potential miR-149 targeting sites. **(D)** Luciferase reporter assay was performed to measure the luciferase activities. **(E, F)** The association among SLCO4A1-AS1, miR-149, and Ago2 was determined using RIP assays in MKN45 cells transfected with miR-149 mimic or inhibitor. **(G)** RT-qPCR analysis of miR-149 level in MKN45 and AGS cells transfected with siSLCO4A1-AS1 or SLCO4A1-AS1 overexpression vector. **(H)** RT-qPCR analysis of miR-149 levels in GC and normal samples; correlation analysis of miR-149 and SLCO4A1-AS1 expression by Pearson’s correlation. **(I)** The expression of miR-149 in 372 GC and 32 normal samples in the ENCORI database; correlation analysis of miR-149 and SLCO4A1-AS1 expression is also shown. *P < 0.05, ^**^ *P* < 0.01.

Several studies have identified that some lncRNAs are likely to function as ceRNAs for miRNAs. Using a bioinformatics analysis tool MiRanda, more than one hundred miRNAs were predicated as possible targets of SLCO4A1-AS1. Of these miRNAs, miR-149-5p, miR-150-5p, miR-193a-5p, miR-331-3p, miR-612, miR-622, miR-637, and miR-1182 have been reported to repress GC cell proliferation and invasion and were selected for further analysis. By SLCO4A1-AS1 pull-down experiments, we purified the SLCO4A1-AS1-associated RNAs and analyzed the 8 candidate miRNAs in the complex, and the result showed a specific enrichment of SLCO4A1-AS1 and miR-149-5p as compared to the controls, whereas the other miRNAs had low enrichment, suggesting that miR-149-5p is the SLCO4A1-AS1-associated miRNA in GC cells (**Figures 2A, B**). To explore the potential correlation between SLCO4A1-AS1 and miR-149, we performed dual-luciferase reporter and RIP assays. We constructed SLCO4A1-AS1 luciferase plasmids including the wild-type (WT) and mutant (MUT) miR-149 binding sites and co-transfected the luciferase plasmids with miR-149 mimics or negative controls into MKN45 and AGS cells (**Figure 2C**). The dual-luciferase reporter assay results indicated that miR-149 overexpression drastically reduced the luciferase activity of the SLCO4A1-AS1-WT vector but did not affect that of the SLCO4A1-AS1-MUT vector (**Figure 2D**). RIP assay results showed that both SLCO4A1-AS1 and miR-149 were enriched after immunoprecipitation using the anti-Ago2 antibody (**Figures 2E, F**). Moreover, RT-qPCR showed that the deletion of SLCO4A1-AS1 expression caused an increase in miR-149 expression, while overexpression of SLCO4A1-AS1 had no obvious effect on SLCO4A1-AS1 level (**Figure 2G**). Furthermore, a decreased miR-149 expression was observed in the GC tissues, and it was negatively correlated to SLCO4A1-AS1 levels (**Figure 2H**). Similar results were obtained using TCGA data of the GC and normal tissues from ENCORI (**Figure 2I**). These findings revealed that SLCO4A1-AS1 directly binds to miR-149 in GC.

### SLCO4A1-AS1 Promotes XIAP Expression by Sponging miR-149

**Figure 3:**
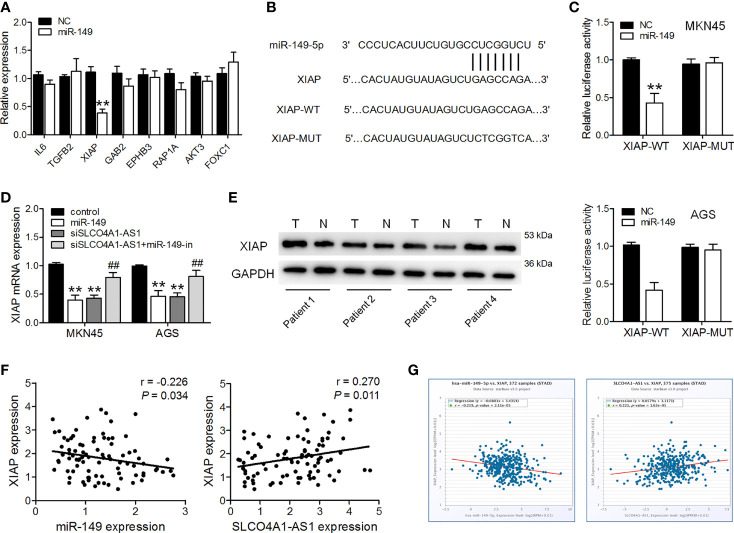
SLCO4A1-AS1 increased XIAP expression by sponging miR-149. **(A)** RT-qPCR analysis of possible targets of miR-149 in MKN45 cells transfected with miR-149 mimic or NC mimic. **(B)** Sequence alignment of XIAP 3′UTR with WT *versus* MUT potential miR-149 targeting sites. **(C)** Luciferase reporter assay was performed to measure the luciferase activities. **(D)** RT-qPCR analysis of XIAP levels in MKN45 and AGS cells transfected with miR-149 mimics, shSLCO4A1-AS1, or both shSLCO4A1-AS1 and miR-149 inhibitor. **(E)** The expression of XIAP in GC and normal tissues was determined using western blotting assay. **(F)** Correlation analysis of XIAP and miR-149 levels in GC samples using Pearson’s correlation; correlation analysis of XIAP and SLCO4A1-AS1 expression in GC tissues. **(G)** Correlation of miR-149 and XIAP expression, and correlation of SLCO4A1-AS1 and XIAP expression are also shown. ^**^ *P* < 0.01 *vs.* control group, ^##^ *P* < 0.01 *vs.* shSLCO4A1-AS1 group.

Using miRDB analysis, we identified 257 candidate genes that were commonly predicted to be possible targets of miR-149-5p (Target score > 70). We did further screening, and genes classified as having molecular functions involved in cell proliferation, migration and invasion were identified. Among them, genes that have roles in cell proliferation, migration and invasion, including IL6, TGFB2, XIAP, GAB2, EPHB3, RAP1A, AKT3, FOXC1, SRPK1, were selected for further analysis. Subsequently, miR-149-5p mimics were transfected into MKN45 cells, and RT-qPCR was used to analyze the expression of these candidate genes. We found that XIAP was the most down-regulated gene in MKN45 cells after miR-149-5p mimics transfection (**Figure 3A**). Furthermore, ectopic expression of miR-149 resulted in a decrease in the luciferase activity of the XIAP 3′UTR-WT vector but did not affect that of the XIAP 3′UTR-MUT vector (**Figures 3B, C**). Overexpression of miR-149 drastically decreased XIAP expression in GC cells (**Figure 3D**). SLCO4A1-AS1 knockdown also lowered XIAP expression, and this suppressive effect was inhibited by an miR-149 inhibitor (**Figure 3D**). Furthermore, XIAP expression was upregulated in the GC tissues, and it was negatively and positively correlated to the miR-149 levels and SLCO4A1-AS1, respectively, in the GC tissues (**Figures 3E, F**). Consistently, we obtained similar results on the negative relationship between miR-149 and XIAP expression, and the positive relationship between SLCO4A1-AS1 and XIAP expression in the GC tissues from the ENCORI database (**Figure 3G**). These data demonstrated that SLCO4A1-AS1 increased XIAP expression through the competitive binding of miR-149 in GC.

### SLCO4A1-AS1 Facilitates GC Cell Proliferation and Invasion *via* the miR-149/XIAP Axis

**Figure 4:**
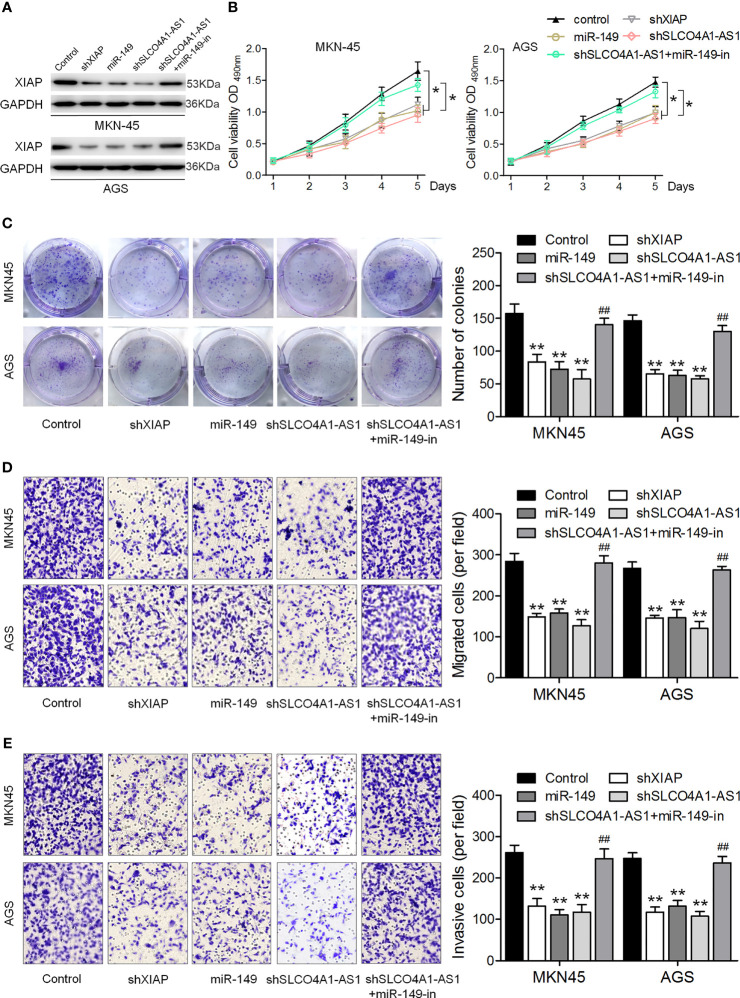
SLCO4A1-AS1 knockdown inhibits the migratory and invasive abilities of GC cells by promoting miR-149 expression. **(A)** Western blotting assay for determining XIAP protein expression in XIAP shRNA, miR-149 mimic, SLCO4A1-AS1 shRNA, and SLCO4A1-AS1 shRNA plus miR-149 inhibitor transfected GC cells. **(B)** Cell proliferation was determined using CCK-8 assay. **(C)** Cell cloning capability was measured using colony formation assay. **(D)** Cell migratory ability was detected using Transwell migration assay. **(E)** Cell invasive ability was determined using Transwell invasion assay. ^*^ *P* < 0.05, ^**^ *P* < 0.01 *vs.* control group. ^##^ *P* < 0.01 *vs.* shSLCO4A1-AS1 group.

**Figure 5:**
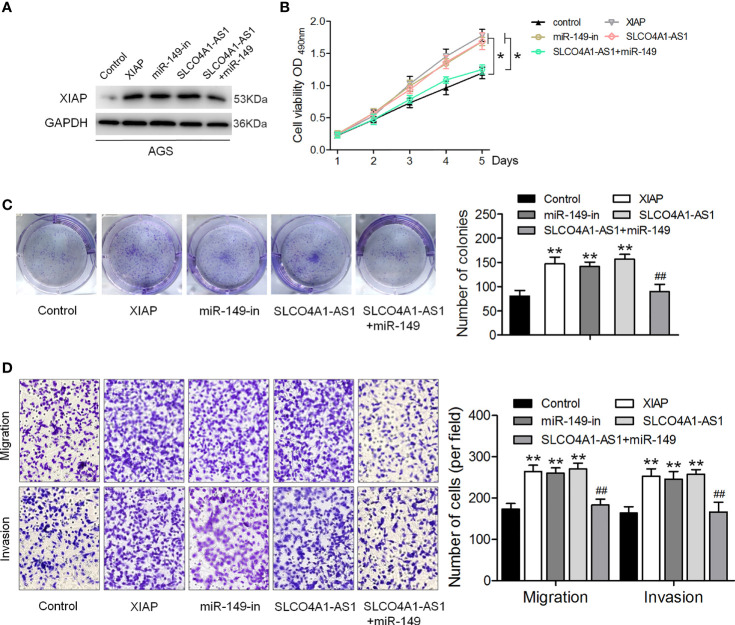
SLCO4A1-AS1 accelerates the migratory and invasive abilities of GC cells *via* the miR-149/XIAP axis. **(A)** Western blotting assay for determining XIAP protein expression in XIAP, miR-149 inhibitor, SLCO4A1-AS1, and SLCO4A1-AS1 plus miR-149 mimic transfected GC cells. **(B)** Cell proliferation was determined using CCK-8 assay. **(C)** Cell cloning capability was measured using colony formation assay. **(D)** Cell migratory and invasive abilities were measured using Transwell assays. ^*^ *P* < 0.05, ^**^ *P* < 0.01 *vs.* control group. ^##^ *P* < 0.01 *vs.*SLCO4A1-AS1 group.

We further investigated the biological function of the SLCO4A1-AS1/miR-149/XIAP axis in GC cells. shXIAP, miR-149, shSLCO4A1-AS1 along with the miR-149 inhibitor were transfected into MKN45 and AGS cells *via* lentivirus transduction, and western blotting assay was conducted to determine XIAP expression (**Figure 4A**). The CCK-8, colony formation, and Transwell assays were then performed; the results indicated that XIAP deletion or miR-149 overexpression resulted in reduced proliferative, invasive, and migratory abilities (**Figures 4B–E**). SLCO4A1-AS1 knockdown also suppressed proliferation, migration, and invasion, whereas these changes were countered by the miR-149 inhibitor (**Figures 4B–E**). Furthermore, AGS cells were transfected with XIAP, miR-149 inhibitor, and SLCO4A1-AS1 alone or cotransfected with SLCO4A1-AS1 and miR-149 inhibitor using lentivirus transduction (**Figure 5A**). Overexpression of XIAP or miR-149 inhibitor promoted cell proliferative, invasive, and migratory abilities (**Figures 5B–D**). Additionally, SLCO4A1-AS1 overexpression promoted GC proliferation, migration, and invasion, whereas these changes were attenuated by miR-149 (**Figures 5B–D**). These results suggest that SLCO4A1-AS1 accelerates the biological functions of GC cells by sponging miR-149 to enhance XIAP expression.

### SLCO4A1-AS1 Exerts Its Prometastatic and Proliferation Activity by Modulating the miR-149/XIAP Levels *In Vivo*

**Figure 6:**
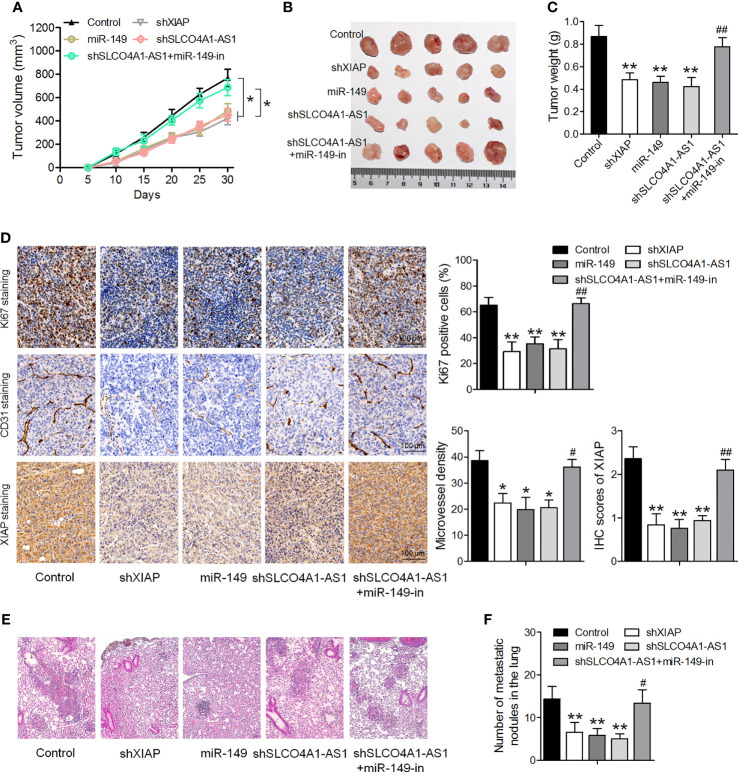
Downregulation of SLCO4A1-AS1 suppresses tumor growth and metastasis *in vivo*. **(A, B)** Nude mice were injected with MKN45 cells infected with XIAP shRNA, miR-149 mimic, SLCO4A1-AS1 shRNA, and SLCO4A1-AS1 shRNA plus miR-149 inhibitor. Tumor growth curves after subcutaneous injection and images are shown. **(C)** Tumor weights. **(D)** Immunohistochemical staining of Ki67, XIAP, and CD31 in the tumors. **(E)** Experimental metastasis in the animal model was conducted by injecting vector-transfected MKN45 cells into the tail vein of the nude mice. Visualization of the H&E-stained lung sections. **(F)** The numbers of tumor nodules on lung surfaces from different groups were observed. ^*^ *P* < 0.05, ^**^ *P* < 0.01 *vs.* control group. ^#^ *P* < 0.05, ^##^ *P* < 0.01 *vs.* shSLCO4A1-AS1 group.

Finally, we sought to determine the role of SLCO4A1-AS1 *in vivo* in GC. Subcutaneous injections of MKN45 cells transfected with shSLCO4A1-AS1, miR-149, and shXIAP or both shSLCO4A1-AS1 and the miR-149 inhibitor were administered to BALB/c nude mice. The tumors formed in the shSLCO4A1-AS1, miR-149, and shXIAP groups were significantly smaller than those in the control group; this effect of shSLCO4A1-AS1 was partly eliminated by treatment with the miR-149 inhibitor 30 days after injection (**Figures 6A, B**). Additionally, the mean tumor weight in the shSLCO4A1-AS1 group was lower; this effect was also partly eliminated by miR-149 inhibitor treatment (**Figure 6C**). Immunohistochemical staining of Ki67 and XIAP showed lower levels of Ki67-positive tumor cells and decreased CD31-positive microvessels and XIAP expression in SLCO4A1-AS1 knockdown tumors (**Figure 6D**). To further assess the function of SLCO4A1-AS1 on GC metastasis *in vivo*, MKN45 cells stably expressing shSLCO4A1-AS1 were injected into the tail vein of the mice. Metastatic nodules on the surface of the lungs appeared after 50 days. As shown in **Figures 6E, F**, knockdown of SLCO4A1-AS1 expression decreased the metastatic nodules on the mice lungs compared with those in the control group. All data indicated that SLCO4A1-AS1 downregulation resulted in the inhibition of GC progression *in vivo*.

## Discussion

Recently, increasing research has revealed the crucial function of lncRNAs in tumorigenesis, including in GC (14). For instance, LINC00703 suppresses GC cell proliferation and invasion but induces apoptosis (15) and lncRNA GMAN promotes hepatocellular carcinoma progression by interacting with eIF4B (16). In the present study, according to the ENCORI database, SLCO4A1-AS1 expression was enhanced in GC samples compared with the normal samples. It was also confirmed that SLCO4A1-AS1 increased in the GC samples and cell lines. SLCO4A1-AS1 knockdown suppresses GC progression by interacting with miR-149 and inhibiting XIAP expression, suggesting that SLCO4A1-AS1 may function as a therapeutic target in patients with GC.

Several studies have shown that a regulation mode exists between lncRNAs and miRNAs, i.e., lncRNAs may negatively regulate miRNA expression level through the regulation mode of ceRNAs and play the role of an endogenous miRNA sponge (17). For example, lncRNA MYOSLID acted as a ceRNA by sponging miR-29c-3p in GC (18). LncRNA MIR17HG facilitates the development of colorectal cancer *via* miR-17-5p modulation (19). This regulatory mechanism is also present in the following lncRNA/miRNAs: MIR210HG/miR-1226-3p (20), LUCAT1/miR-5582-3p (21), and SNHG5/miR-154-5p (22). We inferred that SLCO4A1-AS1 may function as a ceRNA in GC. To strengthen this assertion, bioinformatics analysis was used to explore the potential target of SLCO4A1-AS1. The dual-luciferase reporter assay results supported our hypothesis that miR-149 binds to SLCO4A1-AS1, and the RIP assay further affirmed the participation of RISC in the inhibition process. Moreover, our findings indicated that the downregulation of SLCO4A1-AS1 induced miR-149 expression. Our study also demonstrated that miR-149 overexpression significantly suppressed GC cell proliferation and invasion, which is consistent with previous studies (23, 24). Furthermore, we observed that SLCO4A1-AS1 knockdown inhibited the proliferative, migratory, and invasive capabilities of GC cells whereas these effects were attenuated by the miR-149 inhibitor. These data revealed that SLCO4A1-AS1 increased the biological function of GC cells by sponging miR-149.

The present study further indicated that SLCO4A1-AS1 knockdown decreased XIAP expression. XIAP is a member of the IAP family and has an inhibitory effect on apoptosis (25). Usually, higher levels of XIAP are present in tumor tissues than in normal tissues, and it contributes to tumor cell survival, disease progression, and poor prognosis (26). For example, XIAP could increase MMP2 level to facilitate the progression of bladder cancer (26); moreover, XIAP also promotes the migration of esophageal cancer cells by enhancing epithelial–mesenchymal transition (27). Our findings determined that XIAP was targeted by miR-149, and the overexpression of miR-149 reduced XIAP expression. We also demonstrated that miR-149 directly targeted XIAP 3’UTR to decrease XIAP expression. To investigate whether miR-149 was involved in SLCO4A1-AS1-mediated XIAP expression, various combinations of transfection were used. The results revealed that SLCO4A1-AS1 knockdown combined with the miR-149 inhibitor increased the XIAP levels, suggesting that XIAP has an important function in SLCO4A1-AS1-mediated tumorigenesis. Furthermore, we revealed that downregulated miR-149 expression could reduce the effects exerted by SLCO4A1-AS1 knockdown and demonstrated that SLCO4A1-AS1 increased GC cell proliferation and invasion by sponging miR-149 to upregulate XIAP expression.

In conclusion, we determined that SLCO4A1-AS1 levels are increased in GC tissues and that this upregulation is correlated with disease progression. Furthermore, we indicated that SLCO4A1-AS1 promotes GC proliferation and metastasis by sponging miR-149 to promote XIAP expression, suggesting that SLCO4A1-AS1 functions as a novel prognostic biomarker and a potential antimetastatic therapeutic target for the treatment of GC.

## Data Availability Statement

The raw data supporting the conclusions of this article will be made available by the authors, without undue reservation.

## Ethics Statement

The studies involving human participants were reviewed and approved by the Committee for Ethical Review of Research involving Fudan University Shanghai Cancer Center. The patients/participants provided their written informed consent to participate in this study. The animal study was reviewed and approved by the Committee for Ethical Review of Research involving Fudan University Shanghai Cancer Center

## Author Contributions

YF and BS contributed to the experiment design, manuscript draft, experimental operation, and data analysis. JG collected the clinical data and performed the experiments. YH and CW participated in project design, revised the manuscript, and provided technical support. All authors contributed to the article and approved the submitted version.

## Funding

This study was supported by the National Natural Science Foundation of China (No. 81702347 and No. 81902346).

## Conflict of Interest

The authors declare that the research was conducted in the absence of any commercial or financial relationships that could be construed as a potential conflict of interest.

## Publisher’s Note

All claims expressed in this article are solely those of the authors and do not necessarily represent those of their affiliated organizations, or those of the publisher, the editors and the reviewers. Any product that may be evaluated in this article, or claim that may be made by its manufacturer, is not guaranteed or endorsed by the publisher.
